# Effects of Vitamin B2 Supplementation in Broilers Microbiota and Metabolome

**DOI:** 10.3390/microorganisms8081134

**Published:** 2020-07-27

**Authors:** Elena Biagi, Carlo Mengucci, Monica Barone, Gianfranco Picone, Alex Lucchi, Pietro Celi, Gilberto Litta, Marco Candela, Gerardo Manfreda, Patrizia Brigidi, Francesco Capozzi, Alessandra De Cesare

**Affiliations:** 1 Unit of Holobiont Microbiome and Microbiome Engineering (HolobioME), Department of Pharmacy and Biotechnology (FABIT), Alma Mater Studiorum—University of Bologna, 40126 Bologna, Italy; monica.barone@unibo.it (M.B.); marco.candela@unibo.it (M.C.); 2 Department of Agricultural and Food Sciences (DISTAL), Alma Mater Studiorum—University of Bologna, 40127 Bologna, Italy; carlo.mengucci2@unibo.it (C.M.); gianfranco.picone@unibo.it (G.P.); alex.lucchi3@unibo.it (A.L.); gerardo.manfreda@unibo.it (G.M.); francesco.capozzi@unibo.it (F.C.); 3 DSM Nutritional Products, Basel, Animal Nutrition and Health, Wurmisweg 576, 4303 Kaiseraugst, Switzerland; pietro.celi@dsm.com (P.C.); gilberto.litta@dsm.com (G.L.); 4 Faculty of Veterinary and Agricultural Sciences, The University of Melbourne, Parkville, VIC 3010, Australia; 5 Interdepartmental Centre for Industrial AgroFood Research (CIRI-AGRO), Alma Mater Studiorum—University of Bologna, 47521 Cesena, Italy; patrizia.brigidi@unibo.it; 6 Department of Medical and Surgical Sciences (DIMEC), Alma Mater Studiorum—University of Bologna, 40138 Bologna, Italy; 7 Department of Veterinary Medical Sciences (DIMEVET), Alma Mater Studiorum—University of Bologna, 40064 Ozzano dell’Emilia, Italy; alessandra.decesare@unibo.it

**Keywords:** broiler, microbiota, metabolome, short chain fatty acids, vitamin B2

## Abstract

The study of the microbiome in broiler chickens holds great promise for the development of strategies for health maintenance and performance improvement. Nutritional strategies aimed at modulating the microbiota—host relationship can improve chickens’ immunological status and metabolic fitness. Here, we present the results of a pilot trial aimed at analyzing the effects of a nutritional strategy involving vitamin B2 supplementation on the ileum, caeca and litter microbiota of Ross 308 broilers, as well as on the metabolic profile of the caecal content. Three groups of chickens were administered control diets and diets supplemented with two different dosages of vitamin B2. Ileum, caeca, and litter samples were obtained from subgroups of birds at three time points along the productive cycle. Sequencing of the 16S rRNA V3–V4 region and NMR metabolomics were used to explore microbiota composition and the concentration of metabolites of interest, including short-chain fatty acids. Vitamin B2 supplementation significantly modulated caeca microbiota, with the highest dosage being more effective in increasing the abundance of health-promoting bacterial groups, including *Bifidobacterium*, resulting in boosted production of butyrate, a well-known health-promoting metabolite, in the caeca environment.

## 1. Introduction

Chickens are a high-quality protein source for humans, making poultry one of the most economically valuable animal production systems, a fact which is extremely relevant in a scenario in which food product demand will increase along with the dramatically escalating human population [1,2]. Broilers represent 37% of global meat production, exceeding the bovine and swine industries [3]. In 2019, about 19.7 million metric tons of broiler meat was produced in the United States, and 12.4 million metric tons was produced in the EU [4]. Therefore, it is of the utmost importance for breeders to exploit any possible means available to help in maintaining animal health, and consequently growth performance.

Microbiome science holds great promise for the future of health maintenance and performance improvement in animal production, because gut microbes are responsible for the degradation of complex substrates and energy extraction, as well as for the promotion of the animal’s immune system functionality [1,5]. Indeed, gut microbiome acquisition and maturation are pivotal processes for the development of intestinal epithelium physiology, in terms of immunity, intestinal barrier integrity and nutrient digestion [2,6,7], possibly playing a crucial role in strategies aimed at preventing pathogen colonization and boosting weight gain [8]. Therefore, a key issue in animal production, including chicken nutrition, is to understand the relationship among the effects of diet composition and the changes in microbiota and host metabolism [9].

Chickens’ microbiota is characterized by strong spatial variability along the gastrointestinal tract: specialized communities inhabit different sections of the animal gut, performing specific digestive functions. The most studied of these communities are those residing in the ileum, where nutrient absorption takes place, and the caeca, in which fermentation and digestion of complex polysaccharides occur [10]. The caeca, typical of the avian intestinal tract, are a couple of appendages protruding from the junction of the small and large intestines, in which the feed retention time is the highest, and carbohydrate fermentation, urea recycling and water retention take place [1,8]. Indeed, 10% of the energy recovered from the food is estimated to be produced by fermentative processes occurring in the caeca. In that intestine section, the concentrations of short chain fatty acids (SCFA) and other organic acids (i.e., lactate) are higher than in other tracts [10]. Such microbial products are crucial for host immunological fitness and nutritional homeostasis. Indeed, they provide energy to the epithelial cells, and can be carried to the liver and used as energy substrates for muscle tissue [5]. Some of these compounds can be the subject of microbial cross-feeding, e.g., the lactate produced by *Bifidobacterium* and *Lactobacillus* members can be utilized by other anaerobic bacteria to produce butyrate [11], highlighting the complexity of the microbe–microbe and host–microbe relationships, all involved in defining the final homeostasis and health of the chicken meta-organism.

The microorganisms inhabiting the litter are of both environmental and fecal origin. Litter is continuously pecked and ingested by the animals, thus playing a relevant role in determining the composition of gastrointestinal communities. In addition, litter can act as a reservoir of both animal pathogens and zoonotic agents [1]. Studies in this field pointed out the importance of analyzing changes in the different broilers’ microbiomes over time and how these are affected by intervention strategies to improve animals’ performance. An important focus of such studies must be the effect of interventions on the abundance and persistence of key core microbiota players [12,13,14].

Probiotics and prebiotics are the most accredited strategies to attempt to modify microbiome functionality and composition [15], but other dietary components and nutritional supplements can also modulate gastrointestinal functionality, the gut microbiome, the innate immune system, the intestinal barrier integrity and the intestinal enzyme activity. In this framework, vitamin B2 (riboflavin) can modulate multiple pathways important for the maintenance of the gastrointestinal functionality. There is evidence that vitamin B2 has prebiotic effects [16], affecting the microbiome’s ability to regulate the innate immune system (mucosal associated invariant T cells, MAIT cells). This compound reduces intestinal inflammation and apoptosis and regulates gut protease activity (impacting animals’ food behavior and growth). Moreover, vitamin B2 has been found to be most effective in synergy with antibiotics against methicillin-resistant *Staphylococcus aureus* [17]. Therefore, vitamin B2 can be part of novel solutions that modulate several aspects of gastrointestinal functionality, creating the opportunity to identify additive/synergistic effects with other feed additives.

Here, we report the results of an experimental trial on Ross 308 broilers fed different amounts of vitamin B2. Caeca and ileum microbial communities were longitudinally analyzed along the 42-day broiler productive cycle, together with litter samples, in order to investigate the effects of 50 and 100 mg/kg vitamin B2 dietary supplementation on the microbiota composition and diversity, as well as on the core microbiota components that can persist over time and be shared across the different ecosystems. In addition, in order to explore the supplementation effects on microbial-host co-metabolism, a nuclear magnetic resonance (NMR)-based metabolomics approach was used for analysis of caecal contents.

## 2. Materials and Methods

### 2.1. Animals, Housing and Experimental Diets

The trial was approved by the Ethical Committee of the University of Bologna (Protocol ID 881/2019). Three groups of 120 Ross 308 female chickens each (total number of birds: 360) were housed at the Poultry Research Facility of the University of Bologna in Ozzano dell’Emilia (Italy) in three separate rooms, labelled as A, B and C. The rooms were next to one another and were under identical environmental conditions. Birds reared in each room received a different diet/were fed with a different diet (Room A—control diet; Room B—control diet + 50 mg/kg vitamin B2; Room C—control diet + 100 mg/kg vitamin B2). Each room was divided into three different pens (40 birds/pen). Control diet composition is reported in Table S1. To obtain diets with a medium (Room B) and a high (Room C) level of vitamin B2, the control diet, containing the standard dosage (i.e., 5 mg/kg) of vitamin B2, fitting the recommendations for the whole grow-out phase of broilers fed diets containing wheat (Ross 308 Nutrition Specifications, 2014), was supplemented with vitamin B2 (Rovimix^®^ 80SD; DSM Nutritional Products) up to 50 mg/kg for group B and 100 mg/kg for group C. The dosages in groups B and C (10× and 20× the control, respectively) were set to ensure that a quantity of vitamin B2, largely above the recommended dosage, was able to reach the lower gut. The final content of vitamin B2 in the feed provided to the animals was checked according to the ISO EN 14152:2003 by HPLC (Table S2).

### 2.2. Sampling

Each experimental group was sampled three times: at day 15 (T1), day 28 (T2) and day 42 (T3). During each sampling, a total of 40 birds/room (a total of 120 birds) were randomly selected and euthanized following ethical guidelines to minimize stress and pain. Nine litter samples of 10 g each (3 samples/room; 1 sample/pen) were also collected. The entire gastrointestinal tract was obtained from each bird. Caeca and ileum contents were collected in 2 mL sterile tubes, flash frozen in liquid nitrogen and stored at −80 °C for further investigations. Caeca contents from the 120 birds were collected in duplicate to conduct microbiome and NMR metabolome analyses separately.

### 2.3. DNA Extraction from Caeca, Ileum and Litter Samples

A DNeasy PowerSoil kit (Qiagen, Hilden, Germany) was used for DNA extraction from caeca contents following the manufacturer instructions [14,18]. The protocol used for caeca content was applied to ileal contents with the following modifications to increase DNA yield: (i.) whenever possible, 300 mg of ileal content were used for the DNA extraction, instead of the suggested 200–250 mg; (ii.) elution of the DNA from the Qiagen column was carried out in two steps, using 50 μL each time and incubating the columns for 15 min at 4 °C before each centrifugation. As for litter samples, since the starting material was drier than the intestinal content, the buffer present in the bead tube was not enough to hydrate the 250 mg of litter; thus, 100 μL of sterile physiological solution was added to the samples. The protocol was then carried on as for the caeca samples.

### 2.4. 16S rRNA Gene PCR Amplification and Sequencing

All DNA samples (extracted from caecal, ileal and litter samples) were treated using the same amplification and sequencing protocols. The V3–V4 hypervariable region of the 16S rRNA gene was PCR-amplified using 341F and 785R primers with Illumina overhang adapter sequences, as previously reported [19]. Amplicon purification was performed by using AMPure XP Beads magnetic beads (Beckman Coulter, Brea, CA, USA). For the indexed library preparation, the Nextera XT DNA Library Prep Kit (Illumina, San Diego, CA, USA) was used. A further magnetic bead purification step was performed, and libraries were quantified using the Qubit 3.0 fluorimeter (Invitrogen), then pooled at 4 nM. The library pool was denatured with NaOH 0.2 N and diluted to 6 pM. Sequencing was performed on Illumina MiSeq platform using a 2 × 250 bp paired-end protocol, according to the manufacturer’s instructions (Illumina). Three Illumina sequencing runs were necessary in order to sequence all samples with the appropriate sequencing depth. Care was taken in mixing caeca and ileum samples, as well as samples from the different groups (A, B and C) across the different sequencing runs.

### 2.5. Bioinformatics and Statistics in Microbiota Analysis

Raw sequences were processed using a pipeline combining PANDAseq [20] and QIIME 2 [21] (https://qiime2.org). High-quality reads were filtered and binned into amplicon sequence variants (ASVs) through an open-reference strategy performed with dada2 [22]. The command “qiime dada2 denoise-single” with QIIME 2 version 2019.10 was used with default parameters, with the exception of length filtering (that is already performed by the PANDAseq pipeline). The method used for chimera seq was “pooled”. Taxonomy was assigned using the vsearch classifier [23] and the SILVA database for reference [24]. Alpha diversity was measured using Faith’s phylogenetic distance (PD) index, number of observed ASVs and the Shannon diversity index. Statistics was performed using R Studio software version 1.0.136 running on R software 3.1.3 (https://www.r-project.org/), implemented with the libraries vegan, made4 and PMCMR. Beta diversity was estimated by computing weighted and unweighted UniFrac distances and was visualized by principal coordinates analyses (PCoAs). Bacterial phylogenetic groups showing a minimum relative abundance of 0.5% in at least the 1% of the samples (for each type of sample) were kept for further analysis and graphical visualization. Compositional differences among groups of samples were tested using the Kruskal–Wallis test. P values were corrected for multiple comparisons using the Benjamini–Hochberg method. In addition, bioinformatics analyses were repeated using the QIIME1 pipeline and operational taxonomic unit (OTU) clustering was performed using a 97% similarity threshold and the UCLUST algorithm [25]. This re-analysis allowed for the definition of group of sequences (97%-similarity OTUs) at an intermediate level between genera and species, for which the ecological behavior across the three considered ecosystems (caeca, ileum and litter) was explored as follows. Core 97%-similarity OTUs were identified as those detected with a relative abundance > 0.1% in > 90% of samples in at least 1 time point, as previously reported [14]. Prevalence of the same 97%-similarity OTUs was calculated for all type of samples, at the 3 time points in the 3 groups of broilers (A, B and C), as the percentage of samples in which a given OTU was detected at a relative abundance > 0.1%. The highest score alignment against NCBI 16S rRNA database was obtained by using the BLAST algorithm (https://blast.ncbi.nlm.nih.gov/); identification was limited at the genus level for the majority of the core OTUs, whereas identification at the level of species was considered only when > 99% identity was reached.

### 2.6. Sample Preparation for NMR Analysis

Samples were prepared for NMR analysis by vortex mixing for 5 min stool with 1 mL of deionized water, followed by centrifugation for 10 min at 14,000 rpm at 4 °C. Approximately 540 mL of supernatant was added to 100 μL of a D2O 1.5 M phosphate buffer solution containing 0.1% TSP (3-(trimethylsilyl) propionic acid-d4) and 2 mM NaN3, set at pH 7.40. Before analysis, samples were centrifuged for 10 min again and then 590 μL were transferred into an NMR tube [26].

### 2.7. NMR Spectra Acquisition

Proton NMR (^1^H-NMR) spectra were recorded at 298 K with an AVANCE III spectrometer (Bruker, Milan, Italy) operating at a frequency of 600.13 MHz. The hydrogen deuterium oxide (HOD) residual signal was suppressed by presaturation, whereas broad signals from slowly tumbling molecules were removed by including a Carr–Purcell–Meiboom–Gill filter with a free induction decay sequence. The filter was made up by a train of 400 echoes separated by 800 μs, for a total time of 328 ms. Each spectrum was acquired by summing up 256 transients using 32 K data-points over a 7211.54-Hz spectrum (for an acquisition time of 2.27 s). The recycle delay was set to 8 s, keeping into consideration the longitudinal relaxation time of the protons under investigation. Each spectrum was processed with Top Spin 3.0 (Bruker) by using an automatic command apk0.noe, which performs in one shot the baseline and phase correction, and by applying a line-broadening factor of 1 Hz [27]. The peaks were assigned by comparing their chemical shift and multiplicity with the literature and by using Chenomx NMR suit 8.1 software.

### 2.8. H-NMR Spectra Pre-Processing

After Fourier transformation and baseline correction, spectra were calibrated with reference to the chemical shift of 0.00 ppm assigned to the internal standard TSP; spectral peripheral regions, together with the water signal, were removed. After this, spectra were normalized employing the probabilistic quotient algorithm (PQN) [28] on two different regions separately (regional scaling) since this worked best for this type of sample. After normalization and prior to any possible statistical analysis, spectra were binned into intervals of 100 data-points of 0.0183 ppm each. As a result, the new spectral profile consisted of 410 binned data, which were saved as a matrix in a text file and imported both in R and Python for multivariate statistical analysis (MvSA).

### 2.9. Bioinformatics and Statistics for Metabolomics

All statistical analyses and machine learning routines were carried out in Python 3.6, using implementations from the ScikitLearn package and custom scripts. Ten-fold cross-validation was carried out for each prediction task in order to avoid overfitting. Prediction results reported are the average of the folds.

### 2.10. Partial Least Square Discriminant Analysis (PLS-DA)

Spectra were reduced with ScikitLearn PLSRegression using the NIPALS algorithm [29], modified to suit a discrete classification problem. To select the most important features for each latent variable created, the partial least square (PLS) weights spectra were smoothed with a combination of Savitzky–Golay (SAVGOL) filters [30] and asymmetric least square smoothing and baseline correction [31]. Peaks in weights spectra were furthermore filtered using a signal-to-noise ratio (SNR) threshold, to minimize the probability of selecting uninformative zones of the original NMR spectra.

### 2.11. Classification

Sample group separation was evaluated using the SciKitLearn implementation of C-support vector classifier (SVC) [32]. The parameters for the classifier were estimated using a stochastic grid search, with a linear kernel and a regularization parameter of 0.01 yielding the best performances.

### 2.12. Kinetics Fitting

The term kinetics is hereby used to emphasize the emerging time-dependent variation of the concentrations of the metabolites, of which the estimate was obtained by fitting average concentrations at each one of the three time points. The signals, proportional to the concentration, of metabolites of interest were fitted to highlight possible differences in the variations between treatment groups. For the purpose of the study, the amounts of metabolites are calculated using normalized signal arbitrary units (A.U.), proportional to their molar concentration. Signal distributions were square root transformed, in order to enhance normality and reduce fit bias. At each time point, for each group, metabolite signal was estimated as the average of the signals, while using standard deviation as the error bar for the plots. Time points where fitted using generalized linear models (GLM) from the *statsmodels* (https://www.statsmodels.org/stable/glm.html) module of Python 3.6, with a regression of the form *Y_i_* = α + βlog*X_i_* + ε*_i_*. This allowed us to account for the non-linear relationship between variables, while preserving the linearity of the model and the solution.

A fit confidence interval of 95%, represented by light-colored boundaries in each plot, was reported to assess statistical significance.

The CI was computed using bootstrap resampling [33], to provide an estimate of the variability of the mean tied to the population for each time point, by using the distribution of the means of a sufficiently large number of resamples of the data. This estimation gives an interval where there is a 95% confidence that the true mean of the population lies for each time point. In other words, non-overlapping CI in the plot amongst different treatment groups correspond to statistically different means with *p* < 0.05.

## 3. Results

The aim of this trial was to assess the effects of vitamin B2 supplementation on the ileum, caeca and litter microbiota of broilers, as well as on the metabolic profile of the caecal contents. Therefore, the number of housed birds was not appropriate to calculate production performance indexes. Nonetheless, the average bird body weight was assessed within each group at each sampling time for a comparison with the expected values in relation to the bird ages (Table 1).

### 3.1. Microbial Communities

**Figure 1:**
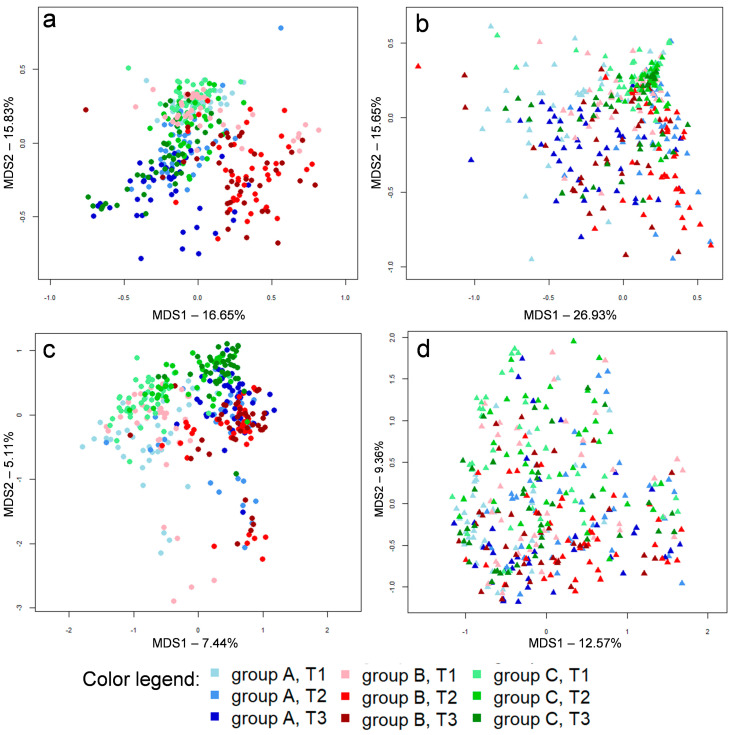
Principal coordinates analyses (PCoA) based on weighted (**a**,**b**) and unweighted (**c**,**d**) UniFrac distances of caecal (**a**,**c**) and ileal (**b**,**d**) microbiota profiles in broilers in groups A (shades of blue), B (shades of red) and C (shades of green). Samples are depicted as dots for caeca and triangles for ileum, filled in different shades of color, from light (earlier samples, day 15, T1) to dark (later samples, day 42, T3), according to the color legend (provided at the bottom). First and second coordination axes are reported in each plot. Percentages of variation in the datasets explained by each axis are reported.

Seven-hundred-forty-one samples were analyzed, including 357 caeca samples, 357 ileum samples and 27 litter samples. For both caeca and ileum, 120 samples from the first time point (14 days, T1), 119 from the second time point (28 days, T2) and 118 from the third time point (42 days, T3) were available. A total of 4,986,865 high-quality sequences were obtained, ranging between 1099 and 15,182, with an average value of 6490 ± 2715 sequences per sample. Sequencing reads were deposited in SRA-NCBI (project number PRJNA644889). Reads were clustered into 20,950 amplicon sequence variants (ASVs). As previously reported [12,14], beta diversity analysis based on both weighted and unweighted Unifrac distances showed a clear separation between ileum and caeca microbial communities, with litter samples clustering in between the two intestinal compartments (Figure S1). In accordance with the available literature [1,14,34], caecal microbiota were consistently dominated by Ruminococcaceae and Lachnospiraceae, whereas in ileal samples Lactobacillaceae was the largely dominant family (Figures S2 and S3). On the contrary, litter samples showed phylogenetic profiles without a clear dominance, with a high abundance and diversity of families belonging to the Proteobacteria and Actinobacteria phyla (Figure S4). Accordingly, litter samples showed higher biodiversity, both measured by Faith’s PD metric (3.17 ± 0.89) and ASV richness (68.96 ± 29.64), with respect to both caecal (Faith’s PD index, 2.37 ± 0.55; ASV richness, 61.32 ± 14.96) and ileal samples (Faith’s PD index, 1.23 ± 0.57; ASV richness, 19.18 ± 9.99). Concerning the Shannon diversity index, litter and caeca samples showed comparable values (4.58 + 0.73 and 4.70 ± 0.47, respectively), both of which were higher than values calculated for ileal samples (2.82 ± 0.62). Beta diversity analysis on available caecal samples (Figure 1a,c) showed that samples taken from group B (supplemented with 50 mg/kg vitamin B2) followed a different longitudinal trajectory in terms of microbiota structure with respect to groups A and C. This trend is particularly evident when weighted Unifrac distances are used to plot the whole sample set (Figure 1a), whereas the PCoA obtained using unweighted UniFrac distances shows more overlap among the different groups (Figure 1c). This indicates that differences in the microbiota of broilers in group B resided in abundant bacterial species, instead of subdominant ones.

**Figure 2:**
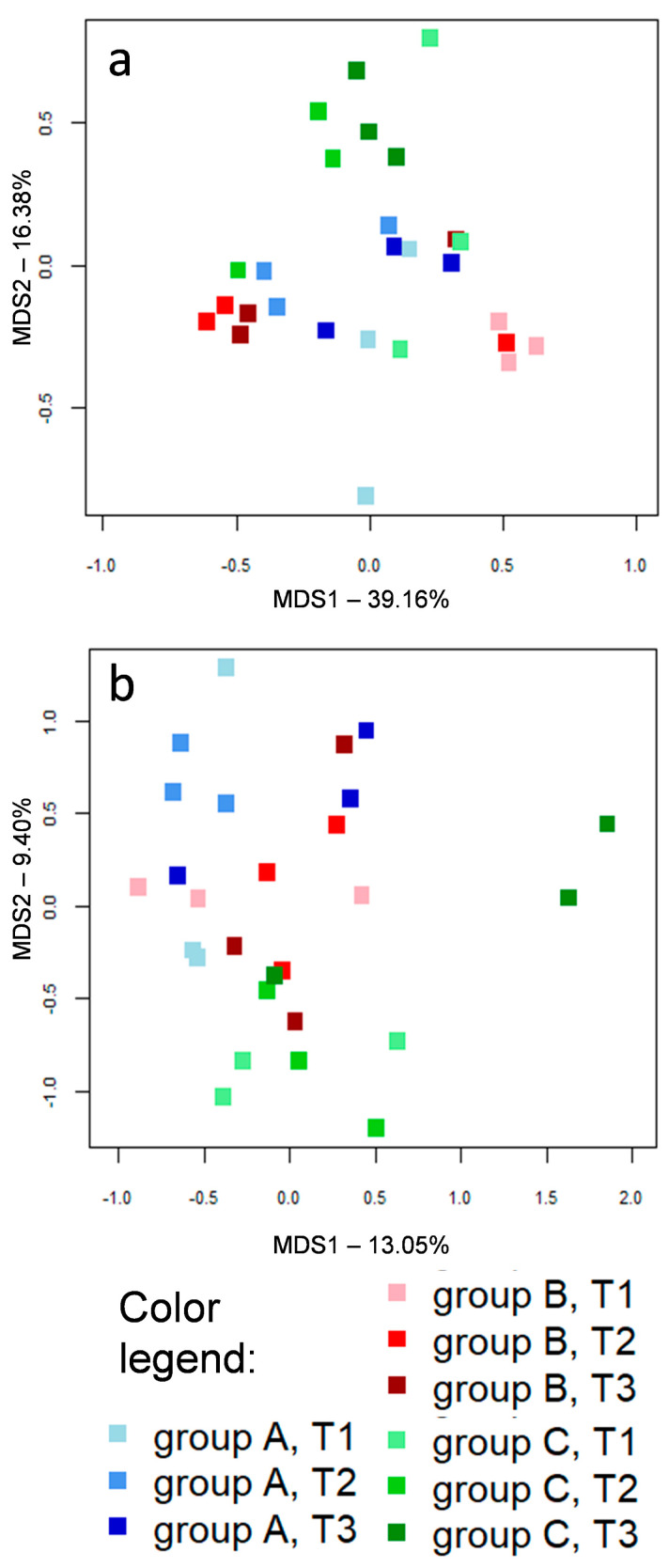
Principal coordinates analyses (PCoA) based on weighted (**a**) and unweighted (**b**) UniFrac distances of litter microbiota profiles in broilers in groups A (shades of blue), B (shades of red) and C (shades of green). Samples are depicted as squares filled in different shades of color, from light (earlier samples, day 15, T1) to dark (later samples, day 42, T3), according to the color legend provided at the bottom. First and second coordination axes are depicted in each plot; percentages of variation in the datasets explained by each axis are reported.

On the contrary, the beta diversity analysis of ileal samples did not show a clear separation across groups A, B and C (Figure 1b,d). Concerning litter samples, PCoA based on weighted and unweighted Unifrac distances are depicted in Figure 2. Samples taken from group C pens showed a slight separation from the other two groups, but this will need to be explored on larger samples.

### 3.2. Caeca, Ileum and Litter Microbiota Composition

**Figure 3:**
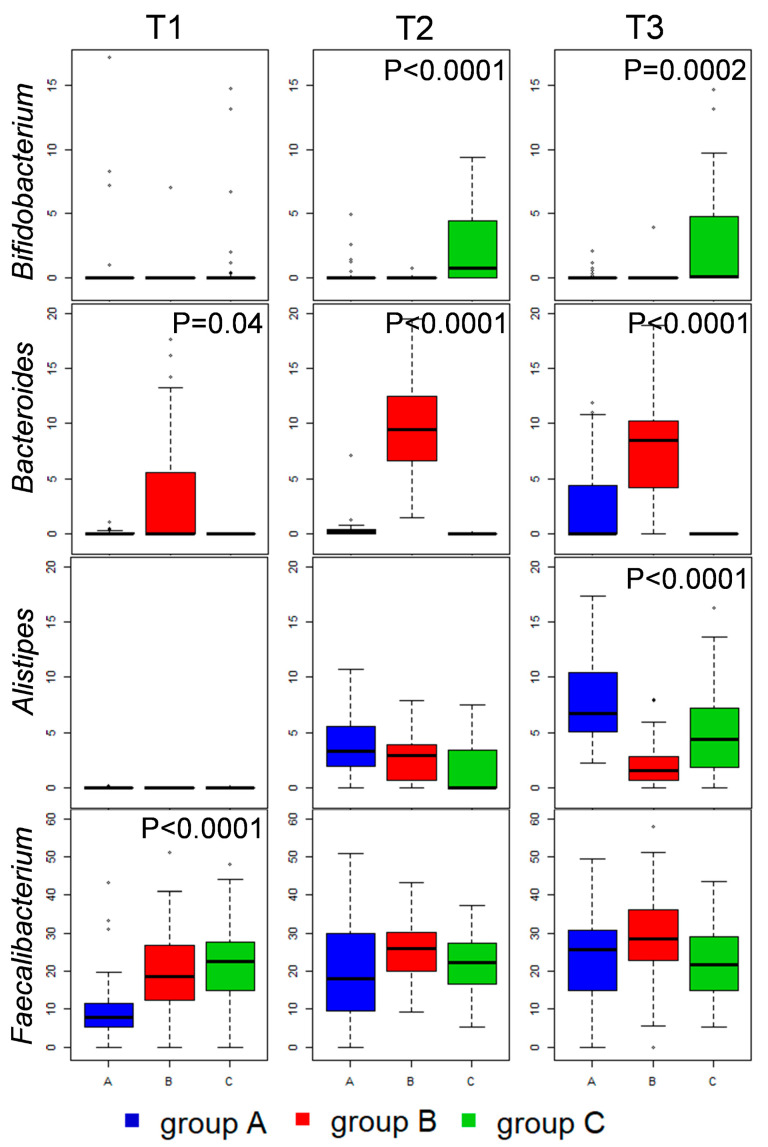
Relative abundance distributions of bacterial genera in the caecal microbiota of broilers. Box and whiskers distributions of relative abundances (%) in all samples at the three time points (from left to right) are depicted for those genera showing significant differences between the three groups (A, blue; B, red; C, green) in at least one time point. Bejamini–Hocherg-corrected *p* values obtained from Kruskal–Wallis test are reported when statistical significance was reached (*p* < 0.05).

The compositional analysis at a family level (Figures S2 and S3) highlighted that supplementation of vitamin B2 (50 mg/kg) (group B) promoted the progressive increase of the Bacteroidaceae family in the caeca, whereas in groups A and C the Bacteroidetes phylum was mostly composed by bacteria belonging to the Rikenellaceae family. This observation was statistically confirmed both at family (Figure S5) and genus levels (Figure 3). Indeed, the family Bacteroidaceae and the genus *Bacteroides* showed significantly higher abundances in group B at all available time points. On the contrary, the family Rikenellaceae and, in particular, its genus *Alistipes* showed higher abundances in groups A and C at T3. Differently, the average family level profiles obtained for the caeca of broilers in group C (supplemented with the highest amount of vitamin B2, 100 mg/kg) showed a progressive increase (from T1 to T3) in the abundance of Bifidobacteriaceae, which was not detected in groups A and B (Figure S5). The significance of this difference was confirmed also at genus levels (genus *Bifidobacterium*, Figure 3) at T2 and T3. The genus level analysis of caeca profiles also showed that vitamin B2 supplementation, in both groups B and C, accelerated the increase in Ruminococcaceae relative abundance, which was significantly higher in group B and C with respect to the control group A at T1 (Figure S5), reflecting an analogous increase in the Ruminococcaceae genus *Faecalibacterium* (Figure 3). At the following time points (T2 and T3) the relative abundance of Ruminococcaceae and/or *Faecalibacterium* in the three groups was not significantly different.

**Figure 4:**
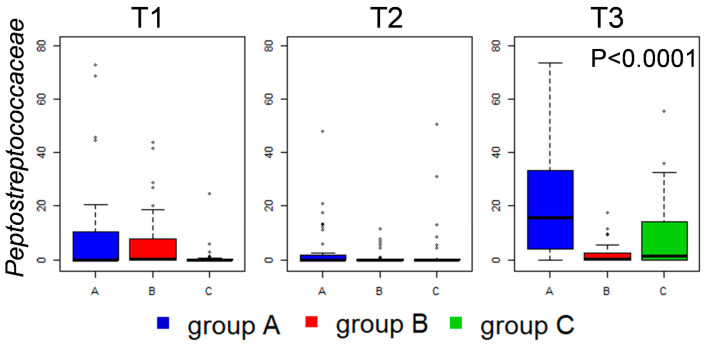
Relative abundance distributions of Peptostreptococcaceae family in the ileum microbiota of broilers. Box and whiskers distributions of relative abundances (%) in all samples, at the three time points (from left to right) are depicted (blue, group A; red, group B; green, group C). Benjamini–Hocherg corrected *p* values obtained from Kruskal–Wallis test are reported when statistical significance was reached (*p* < 0.05).

Ileal microbiota composition was less affected by vitamin B2 supplementation. Compositional analysis only showed that, at T3, both vitamin B2-supplemented diets (groups B and C) significantly inhibited an increase in the abundance of the Peptostreptococcaceae family, which was evident in the control diet (group A) (Figure 4).

### 3.3. Ecological Perspective of Broiler Caeca, Ileum and Litter Microbiota

**Figure 5:**
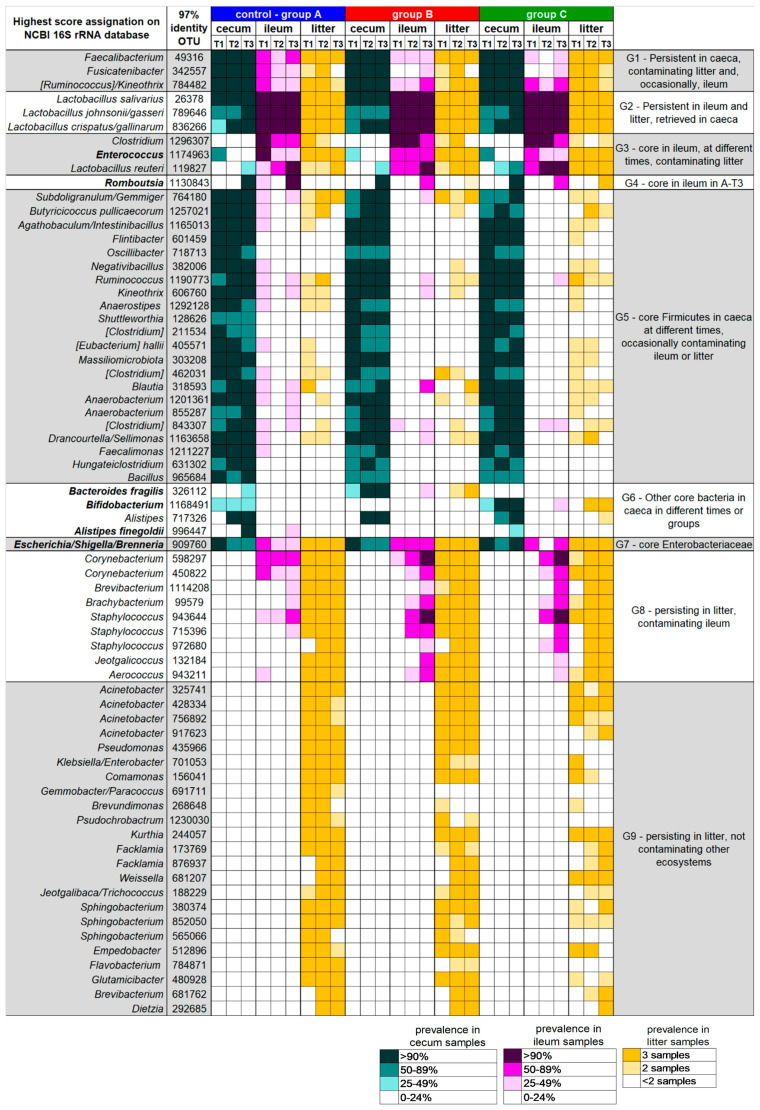
Prevalence of core operational taxonomic units (OTUs) at 97% similarity, in broilers’ caeca and ileum and litter from groups A, B and C, at the three time points (T1, T2, and T3). Operational taxonomic units (OTUs) at 97% similarity were obtained by using the qiime1 pipeline. OTUs detected with a relative abundance > 0.1% in > 90% of samples in at least 1 time point are shown, together with the identification of the highest score alignment against the NCBI 16S rRNA database obtained by using BLAST nucleotide algorithm. Identification is at the level of species only when > 99% similarity was reached, whereas more than one possible genera are reported when equal scores were obtained. Shades of sea-green, purple, and gold are used to indicate the degree of prevalence of the OTUs in all available sets of samples, according to the provided color legend (bottom). OTUs were grouped according to their ecological behavior across the three analyzed ecosystems (caeca, ileum and litter), obtaining groups G1 to G9, as depicted in the right column.

A subsequent re-analysis of the sequences using a different OTU picking strategy (UCLUST algorithm with 97% similarity threshold) was performed to facilitate the interpretation of the ecological behavior of the most prevalent and persistent bacterial groups across the three analyzed ecosystems, as well as to evaluate the impact of vitamin B2 supplementation at an ecological level. Indeed, the 97%-similarity threshold allowed us to obtain groups of sequences, possibly ascribable to small group of species, that could play specific ecological roles within and across the caeca, ileum, and litter microbial ecosystems. Among the obtained 97%-similarity OTUs, we filtered those detected with a relative abundance > 0.1% in > 90% of samples in at least one time point, thus defining ecosystem-specific “core microbiota”. For these “core 97%-similarity OTUs” the prevalence, i.e., the percentage of samples in which each OTU was detected at a relative abundance > 0.1%, was calculated for all available samples and plotted using a color code in the heatmap in Figure 5. The observation of the prevalence of each OTU across the different samples allowed for the clustering of the core OTUs into nine groups.

Group G1 comprised those core bacteria of the caeca that were persistent along the longitudinal sampling, including OTUs assigned to the well-known health-promoting *Faecalibacterium*; vitamin B2 supplementation did not affect the prevalence of these OTUs in the caeca, but seemed to have an impact on their prevalence in the ileum. Group G2 included OTUs assigned to *Lactobacillus* species that were persistently part of the ileum core microbiota, confirming the available literature [14], but are also frequently retrieved from the litter and caeca. Group G3 comprised *Clostridium*, *Enterococcus* and *Lactobacillus* OTUs that were part of the core ileal microbiota only in one or two time points, and only occasionally retrieved from the caeca; *Enterococcus* prevalence in the ileum seemed to be affected by vitamin B2 supplementation. Group G4 included only one 97%-similarity group of sequences, belonging to the Peptostreptococcaceae family and assigned to the genus *Ramboutsia*, that was part of the core ileal and caeca microbiota at T3 in the control group, but less prevalent in groups B and C (62% in both); this taxon could be responsible for the significant decrease in the ileal relative abundance of the Peptostreptococcaceae family associated with vitamin B2 supplementation (Figure 4). Group G5 included Firmicutes members that were part of the caecal core microbiota in at least one time point, occasionally contaminating ileum and litter samples, and that seemed to maintain such ecological behavior independently of vitamin B2 supplementation; several of these OTUs were assigned to genera known for their butyrate-production capability, such as *Subdoligranulum*, *Butyricicoccus*, *Agathobaculum*, *Kineothrix*, and *Anaerostipes* [11,35,36], or their acetate-production capability, like *Blautia* and *Faecalimonas* [37]. On the contrary, group G6 included non-Firmicutes OTUs of the caeca microbiota, of which the prevalence was deeply affected by vitamin B2 supplementation. An OTU assigned to the species *Bacteroides fragilis* was part of the caeca’s core microbiota only at T2 and T3 in broilers supplemented with 50 mg/kg vitamin B2 (group B), reflecting the group B-specific significant increase in abundance of *Bacteroides* (Figure 3 and Figure S5). Concerning the genus *Alistipes*, of the Rikenellaceae family, an OTU putatively assigned by the BLAST algorithm to the species *Alistipes finegoldii* was probably responsible for the significant difference in the relative abundance of *Alistipes* across groups at the last time point (Figure 3), with this genus being part of the caeca core microbiota only in the control group at T3, and being absent in group B. Furthermore, confirming relative abundance data (Figure 3 and Figure S5), a Bifidobacterium-assigned OTU was found in part of the caecal core microbiota only in group C broilers, at T2 and T3. The G7 group included only one OTU belonging to the Enterobacteriaceae family (possibly assigned to the *Escherichia/Shigella* group) that consistently colonized the litter, but also the caeca and ileum. The last two groups of OTUs, G8 and G9, included bacteria prevalently colonizing the litter. OTUs included in group G8 are occasionally found in ileum samples, which they could possibly reach through litter ingestion by the broilers. It is possible to notice that in broilers receiving the highest amount of vitamin B2 (group C) the persistence and prevalence of G9 OTUs was lower, possibly explaining the slight separation observed during the beta diversity analysis using unweighted Unifrac distances (Figure 2b).

### 3.4. Caeca Metabolome Analysis

**Figure 6:**
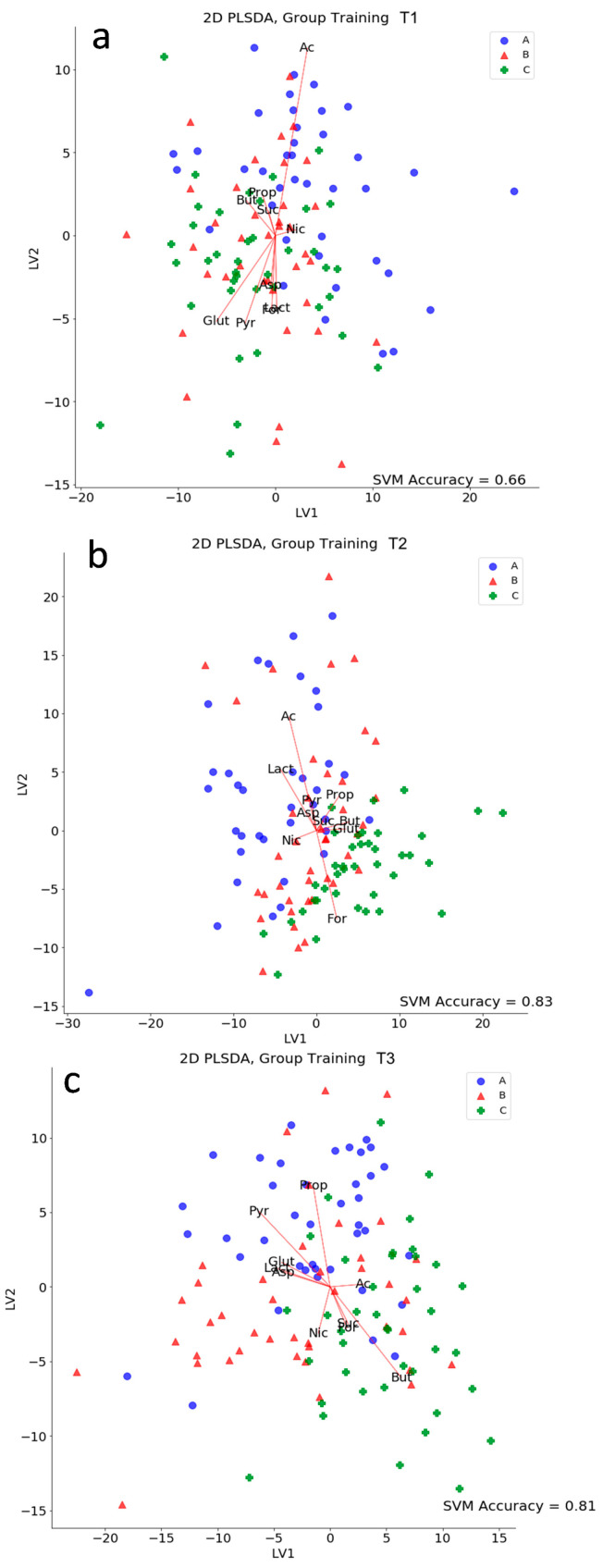
Partial least square score plots at time points T1 (**a**), T2 (**b**), and T3 (**c**). The support vector machine classifier accuracy score is reported at each time point in order to highlight at which time the effects of the treatment are more detectable in the metabolic space (T2). Red arrows mark the directions of maximum expression in the metabolic space for each metabolite of interest. Points scattered along a particular direction are expected to be characterized by an abundance of the related metabolite. Ac: acetate, Pyr: pyruvate, Asp: aspartate, Lact: lactate, Nic: nicotinate, For: formate, Glut: glutamate, But: butyrate, Suc: succinate, Prop: propionate.

A total of 357 spectra were used to train a PLS-DA for spectral dimensionality reduction. This served as a projection of the whole NMR spectrum to a lower dimensional metabolic space, with the aim of enhancing visualization and finding possible clusters. Preliminary unsupervised analyses showed that animal growth (time) was the most influential factor in metabolic changes, thus being the biggest cause of sample separation in multivariate analysis. PLS-DA was then carried out for all samples at each time point, with the task of discriminating between treatments (Figure 6). The best separation between groups, evaluated using SVC accuracy of prediction, was obtained at T2 (Figure 6b). At this time point, animals seemed to show the highest metabolic response to treatment. This points out the time window in which treatment effects are the most detectable from a metabolic perspective.

### 3.5. Kinetics of Relevant Metabolites

**Figure 7:**
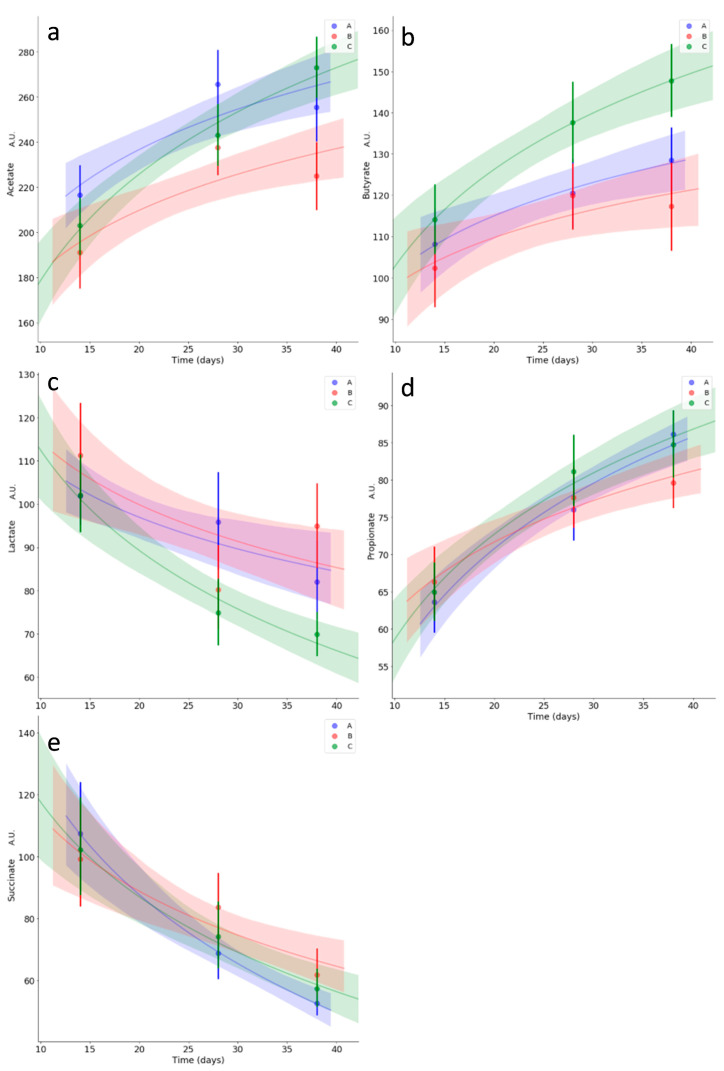
Kinetics for caecal concentration of acetate (**a**), butyrate (**b**), lactate (**c**), propionate (**d**) and succinate (**e**). Average values for treatment groups A, B and C are reported in blue, red and green, respectively. Translucent bands represent each fit’s 95% confidence interval. Zones of the fits with non-overlapping bands correspond to statistically meaningful differences in trends between groups. Significant differences in trend are seen for acetate, butyrate and lactate. Metabolites are reported using normalized signal arbitrary units (A.U.), proportional to concentration.

**Figure 8:**
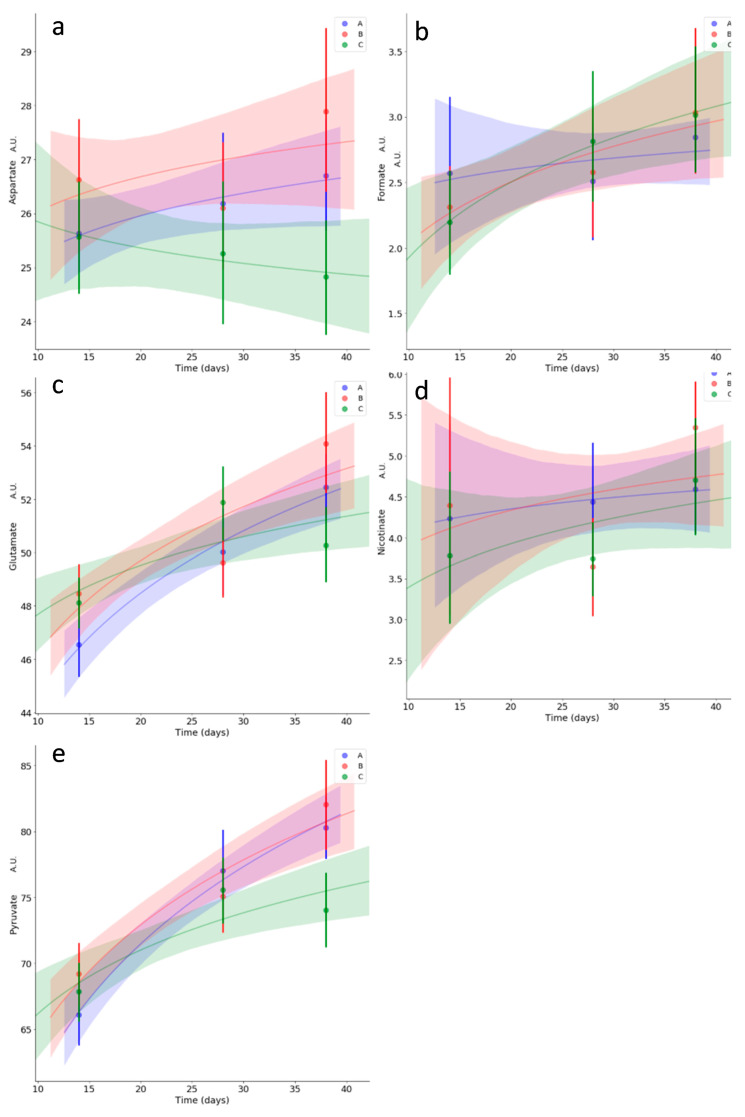
Kinetics for caecal concentration of energy metabolism related metabolites: aspartate (**a**), formate (**b**), glutamate (**c**), nicotinate (**d**) and pyruvate (**e**). Average values for treatment groups A, B and C are reported in blue, red and green, respectively. Translucent bands represent each fit’s 95% confidence interval. Zones of the fits with non-overlapping bands correspond to statistically meaningful differences in trends between groups. Only pyruvate started to show a different late trend for group C with respect to the other treatments. Metabolites are reported using normalized signal arbitrary units (A.U.), proportional to concentration.

Kinetics studies were carried out on two categories of metabolites of interest, i.e., short chain fatty acids and energy metabolism-related metabolites. Nominally, acetate, propionate, lactate, succinate and butyrate were selected for the first category (Figure 7), and aspartate, glutamate, nicotinate, formate and pyruvate were selected for the second (Figure 8). Treatment B had a significant dampening effect on acetate starting from day 28, whereas group A and C trends remained similar (Figure 7a). The butyrate trend was an overall increase over time, with a statistically significant increase for treatment group C starting from day 28 (Figure 7b). Lactate had an overall decreasing trend, with group C decreasing significantly faster (Figure 7c). Pyruvate showed an overall increasing trend over time, with a statistically significant late dampening effect given by treatment C (Figure 8e). Aspartate, formate, nicotinate, glutamate, propionate and succinate showed no statistically meaningful differences in trends for the treatment groups (Figure 7d,e; Figure 8a–d). Aspartate showed overall high variability, along with nicotinate. Formate and propionate increased over time with a similar trend for all the treatment groups, whereas succinate decreased over time with a similar trend for all the treatment groups.

## 4. Discussion

The intestinal microbiota of homoeothermic animals constitutes a complex ecosystem composed of a large variety of microorganisms. It plays an important role in maintaining the host’s normal gut functions and health, and its imbalance, or dysbiosis, can produce negative effects on gut physiology [38]. Since the ban of antibiotics as growth promoters in the European Union [39], alternative strategies to improve broilers’ immunological and metabolic fitness are of great interest. Those strategies involve manipulation of the host–microbiota relationship, through administration of dietary components as well as pro/prebiotics [40,41]. Although not providing a direct substrate for microbial fermentation, riboflavin was reported to influence the gastrointestinal redox state, ultimately modulating the composition of the intestinal microbiota towards an advantageous configuration [42]. In the present study, the effects of supplementation of different dosages of vitamin B2 were studied at a model scale.

Vitamin B2 supplementation did not affect the ecosystem specificity of the microbial communities, since sample type (caeca, ileum, and litter) remained the main driver of bacterial composition, as previously noted [12,14]. However, the treatment was able to exert a specific effect on both caeca and ileum microbiota components, affecting different bacterial groups and influencing the caecal concentration of different metabolites, depending on the vitamin dosage. Even though the dosage effect has a biological base, the authors cannot exclude the possibility that differences in microbiota components and metabolites are due to a room effect rather than a treatment effect. Therefore, this variable will be taken into account in future studies.

Confirming previous reports on the effect of vitamin supplementation on broiler caecal microbiota [43], both vitamin B2 dosages (i.e., 50 and 100 mg/kg) induced an increase of the well-known health-promoting bacteria belonging to the genus *Faecalibacterium* [12] during the first two weeks of the broiler’s productive cycle (T1). Moreover, both vitamin B2 dosages also reduced the progressive increase in Rikenellaceae that was observed, through T1 to T2 to T3, in the control group. Indeed, our data showed that OTUs assigned to the species *Alistipes finegoldii* appeared at T3 in the caecal core microbiota of more than 90% of broilers in the control group, whereas this did not happen in broilers in group B and C. This bacterial species had previously been associated with a low food conversion rate (FCR) in broilers [44], whereas *Faecalibacterium* was reported to be positively correlated with FCR, as well as other productivity parameters [14,44,45].

The highest concentration of vitamin B2 (group C) induced an increase in the abundance of a well-known health-promoting group of lactic acid producers whose genetic makeup lacks enzymes needed for the biosynthesis of this vitamin (*Bifidobacterium*) [46]. Interestingly, metabolomics analysis highlighted a progressive decrease of lactate in group C, in favor of butyrate accumulation. This could be explained by the fact that lactate is not usually accumulated in the gut environment, but is consumed as a result of metabolic cross-feeding between lactate-producing and lactate-utilizing bacteria, some of which can use it as a precursor for butyrate synthesis [11]. Indeed, the highest dosage of vitamin B2 (group C) seemed to be the one promoting a microbial co-metabolism, leading to a final increased concentration of butyrate, although no significant increase in the abundance of well-known butyrate producers was detected at later time points.

On the contrary, the intermediate concentration of the vitamin (group B, 50 mg/kg) significantly increased the *Bacteroides* abundance in the caeca along the whole productive cycle, with the appearance of an OTU assigned to *Bacteroides fragilis* in the core caecal microbiota. As previously reported [47], the *Bacteroides* increase was to the detriment of the family Rikenellaceae (*Alistipes*), also member of the phylum Bacteroidetes. According to a review of the literature, Bacteroidaceae and Rikenellaceae abundances in broilers’ guts is strongly influenced by dietary supplements and ingredients [47,48,49,50,51,52], and the species *B. fragilis* was already indicated as responding to changes in dietary regimen in broilers [53]. Most importantly, an increase in the caecal abundance of *Bacteroides*, and/or the species *B. fragilis*, had already been associated with body weight gain and improved performance [14,54]. The observed changes in microbiota taxonomy in group B were not mirrored at the metabolomics level. Indeed, propionate, which is a common terminal fermentation product of Bacteroidetes, did not increase in the caeca content. This apparent inconsistency may be explained by a subsequent conversion of propionate at higher rates than its increased production from Bacteroidetes, resulting in a null effect on the steady-state concentration of such metabolites.

Looking at proxy metabolites of the energetic metabolism, the only one showing a statistically significant difference with respect to the treatment groups was pyruvate, starting from the late stage of the fitted model for group C. Pyruvate’s increasing trend in caeca samples was lower in group C with respect to the other two groups. This may be due to the fact that the energy production progressively decreases with age in all organisms, mainly due to the decline in the function of mitochondria [55]. In chickens, such disrupted homeostasis may lead to an increased excretion of involved metabolites and their consequent increasing appearance in the excretory apparatus. Conversely, high doses of vitamin B2 might positively affect the age-related impairment of energy metabolism by slowing it down. Another possible explanation could be related to an increased transformation of pyruvate into butyrate, via Acetyl-CoA intermediate production, operated by members of the Ruminococcaceae family [56].

Concerning the ileal microbiota, both diets administered to broilers in groups B and C had a marginal impact on microbial composition. However, it was possible to appreciate that vitamin supplementation counteracted the physiological increase in Peptostreptococcaceae, in particular the increase of an OTU putatively assigned to the genus *Ramboutsia*, a slow-growing taxon known to be detected in the later developmental stage of the ileal microbiota assembly [57,58]. The ecological significance of this taxon still has to be explored.

Litter is frequently analyzed in studies focused on the microbiome of broilers because, even if the population structure is very different from the ones found in the animals’ gut, some groups of bacteria are shared, and changes in litter microbiota were associated with changes in flock productivity [2,7,59]. Furthermore, litter can act as a reservoir of bacteria that enter the environment, possibly impacting environmental microbiome composition [60] and spreading zoonosis and antimicrobial resistance. Core litter microbiota in our study were consistent with previous reports [14], being partly composed of bacteria abundant in the caeca and ileum, such as *Faecalibacterium*, *Enterococcus*, *Lactobacillus* and *Enterobacteriaceae*, assigned to the *Escherichia*/*Shigella* group. Ileum and litter core microbiota also shared several Actinomycetales and Bacillales taxa, belonging to the genera *Corynebacterium*, *Brevibacterium*, *Brachybacterium*, *Staphylococcus* and *Jeotgalicoccus* [61], but they tended to appear in the ileum at T2 and T3, indicating that they probably contaminated the animals’ guts through litter ingestion. Moreover, litter core microbiota were characterized by a group of taxa, mostly belonging to the Proteobacteria phylum, the presence and persistence of which seemed to be diminished by vitamin B2, especially with the highest dosage, highlighting a possibly positive effect of vitamin supplementation on the environmental microbiome circulation during waste management.

In conclusion, the supplementation of 50 and 100 mg/kg of vitamin B2 was effective in modulating the composition of caeca microbiota, with a marginal impact also on ileal community structure. In particular, the supplementation of vitamin B2 at 50 mg/kg significantly increased the *Bacteroides* abundance since day 14 up to the end of the rearing cycle. Moreover, the highest dosage of vitamin B2 (100 mg/kg) significantly increased the abundance of *Bifidobacterium* starting from day 28 up to 42 days. This microbiota modulation resulted in the boosted production of butyrate, which plays an important role in protection against pathogens in poultry [62]. Furthermore, butyrate is involved in several intestinal functions, being an energy source stimulating epithelial cell proliferation and differentiation, other than exerting an antimicrobial effect by promoting the production of peptides and stimulating the production of tight junction proteins [63]. Therefore, the proposed nutritional integration could positively affect the host’s fitness in reacting to pathogenic infections through a butyrate-mediated improvement of epithelial integrity in the caeca and positive stimulation of the immune system.

## Figures and Tables

**Table 1:** Body weight (g) of Ross 308 female broiler chickens at different age of life in relation to different groups. Total number of birds that survived the whole trial is reported for each group.

Group	n. of Chickens	Chicken Weight (g) (Mean ± SD)		
		14 days	28 days	42 days
Group A—control 5 mg/kg VitB2	120	528.78 ± 8.48	1431.95 ± 15.56	2619.85 ± 22.10
Group B—50 mg/kg VitB2	119	532.97 ± 9.36	1433.43 ± 12.63	2622.33 ± 20.51
Group C—100mg/kg VitB2	118	534.46 ± 9.36	1433.15 ± 13.66	2618.92 ± 19.27

SD: Standard deviation.

## Supplementary Materials

The following are available online at https://www.mdpi.com/2076-2607/8/8/1134/s1. Supplementary Figure S1: Principal coordinates analyses (PCoA) based on weighted (a) and unweighted (b) UniFrac distances of caecal (sea-green), ileal (purple) and litter (gold) microbiota profiles. Supplementary Figure S2: Family-level microbiota profiles of broiler caeca ecosystem. Supplementary Figure S3: Family-level microbiota profiles of broiler ileum ecosystem. Supplementary Figure S4: Family-level microbiota profiles of litter ecosystem in broiler groups A, B and C. Supplementary Figure S5: Relative abundance distributions of bacterial families in the caecal microbiota of broilers. Supplementary Table S1: Feed composition (%) in relation to different feeding phases. Supplementary Table S2: Vitamin B2 target and analyzed content in feed for each group, (mg/kg of feed and % vs. target), in the different feeding phase diets.
