# Supplementary material for: Effects of Vitamin B2 Supplementation in Broilers Microbiota and Metabolome
Source: Microorganisms. 2020 Jul 27;8(8):1134. doi: 10.3390/microorganisms8081134 (PMC7464963; doi:10.3390/microorganisms8081134)
Supplement: Supplementary file 1 [file microorganisms-08-01134-s001.pdf]

Effects of vitamin B2 supplementation in broilers microbiota and metabolome

Elena Biagi*, Carlo Mengucci, Monica Barone, Gianfranco Picone, Alex Lucchi, Pietro Celi, Gilberto Litta, Marco Candela, Gerardo Manfreda, Patrizia Brigidi, Francesco Capozzi, Alessandra De Cesare

*elena.biagi@unibo.it

SUPPLEMENTARY MATERIALS

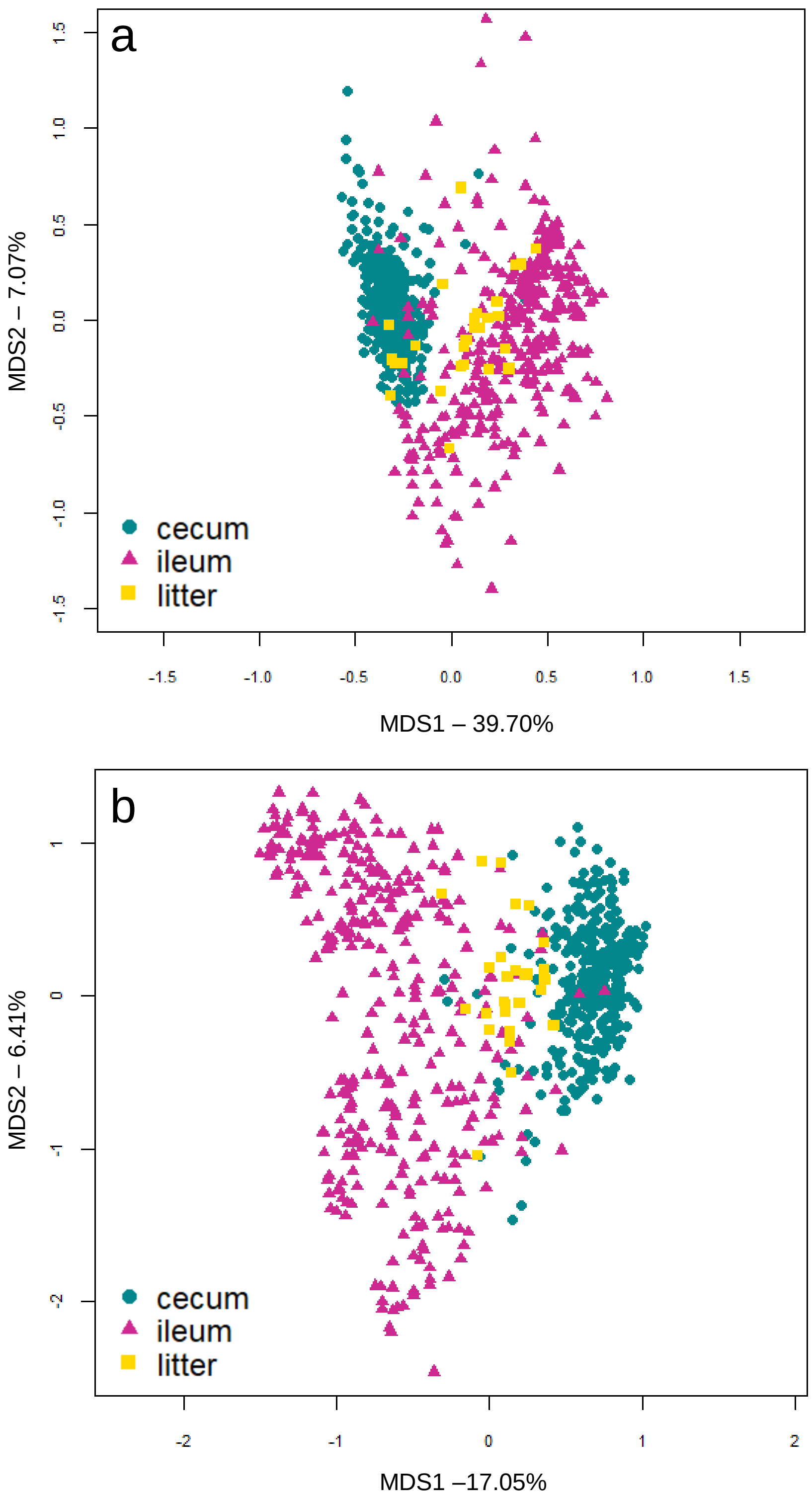

Supplementary Fig. S1: Principal Coordinates Analyses (PCoA) based on Weighted (a) and Unweighted (b) UniFrac distances of caecal (seagreen), ileal (purple) and litter (gold) microbiota profiles. Samples are depicted as dots for caeca, triangles for ileum, and squares for litter, according to the color legend. First and second coordination axes are reported in each plot; percentages of variation in the datasets explained by each axes are reported.

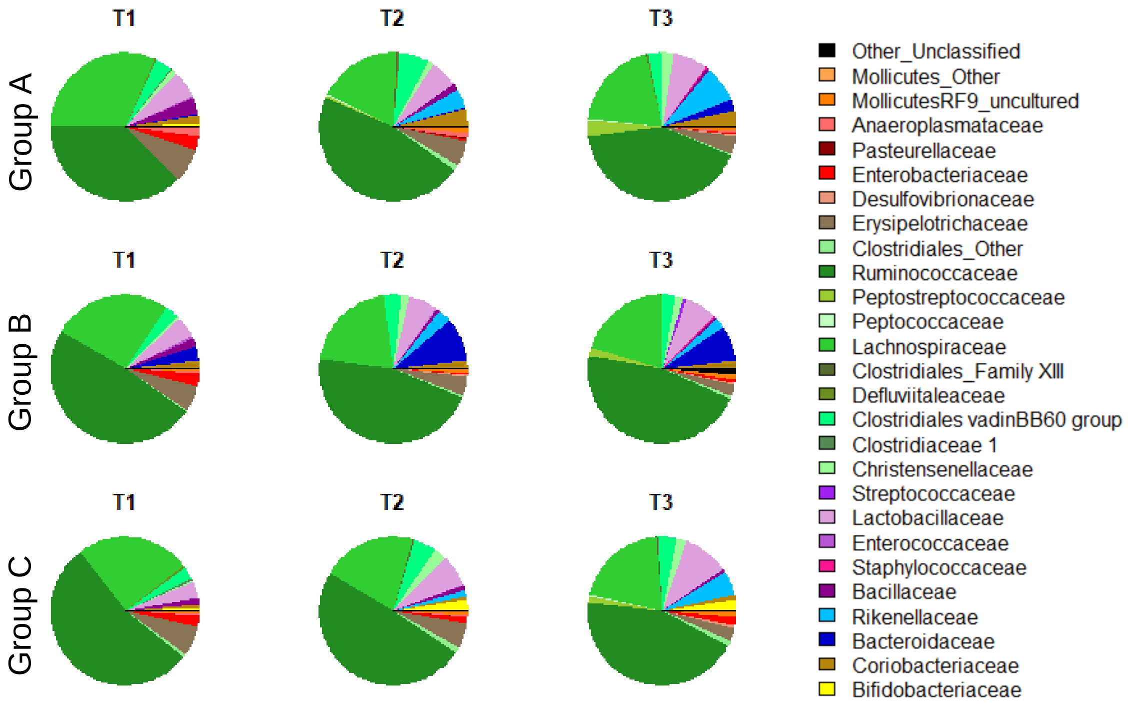

Supplementary Figure S2: Family level microbiota profiles of broiler caeca ecosystem. Average phylogenetic profiles of the caecal microbiome of broilers in groups A, B and C (top-down), for each available time point (T1, T2, T3; from left to right) are provided as pie charts at family level; bacterial taxa were filtered for graphical representation as >0.5% in at least 1% of all the available samples.

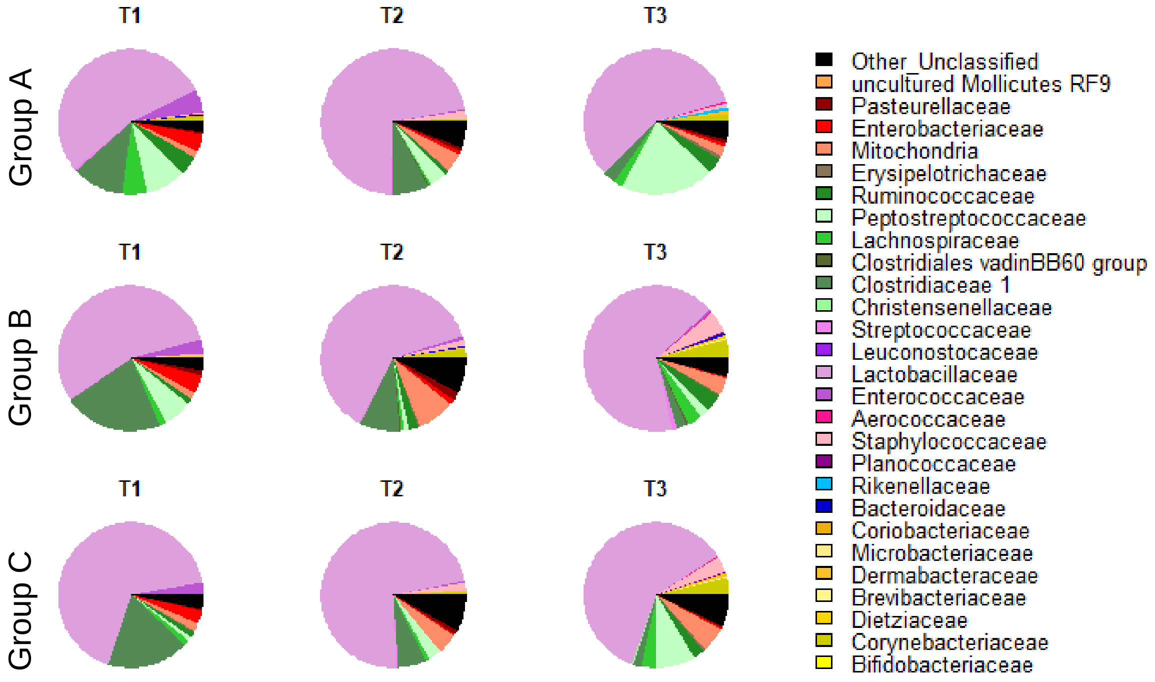

Supplementary Figure S3: Family level microbiota profiles of broiler ileum ecosystem. Average phylogenetic profiles of the ileal microbiome of broilers in groups A, B and C (top-dowm), for each available time point (T1, T2, T3; from left to right) are provided as pie charts at family level; bacterial taxa were filtered for graphical representation as >0.5% in at least 1% of all the available samples.

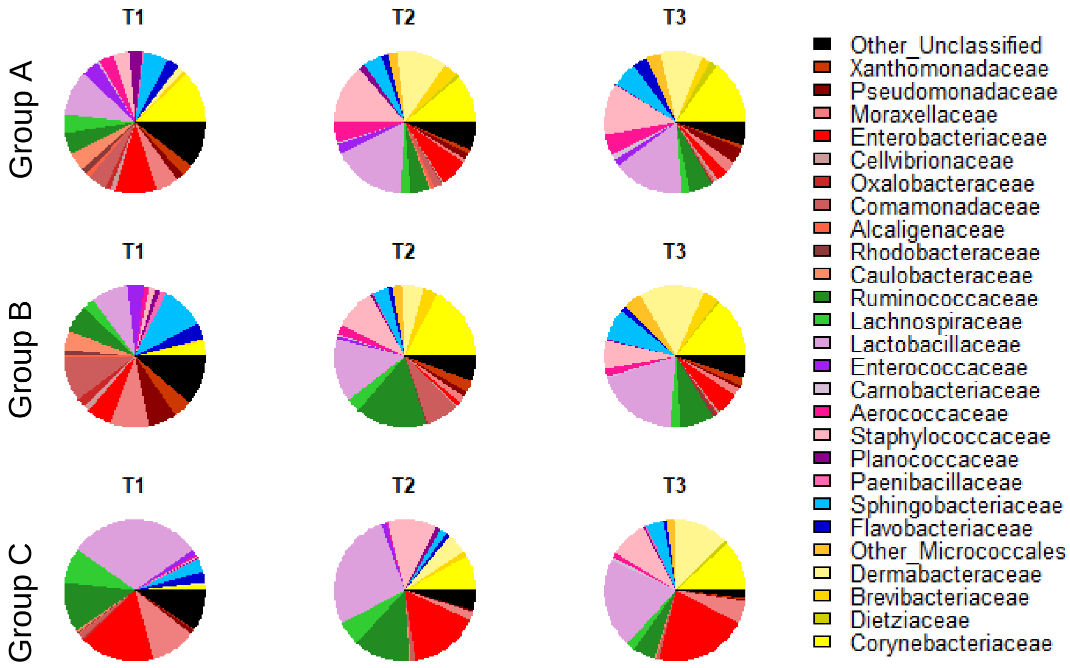

Supplementary Figure S4: Family level microbiota profiles of litter ecosystem in broilers group A, B and C. Average phylogenetic profiles of the litter microbiome in broilers groups A, B and C (top-down), for each available time point (T1, T2, T3; from left to right) are provided as pie charts at family level; bacterial taxa were filtered for graphical representation as >1% in at least 3 out of 27 samples.

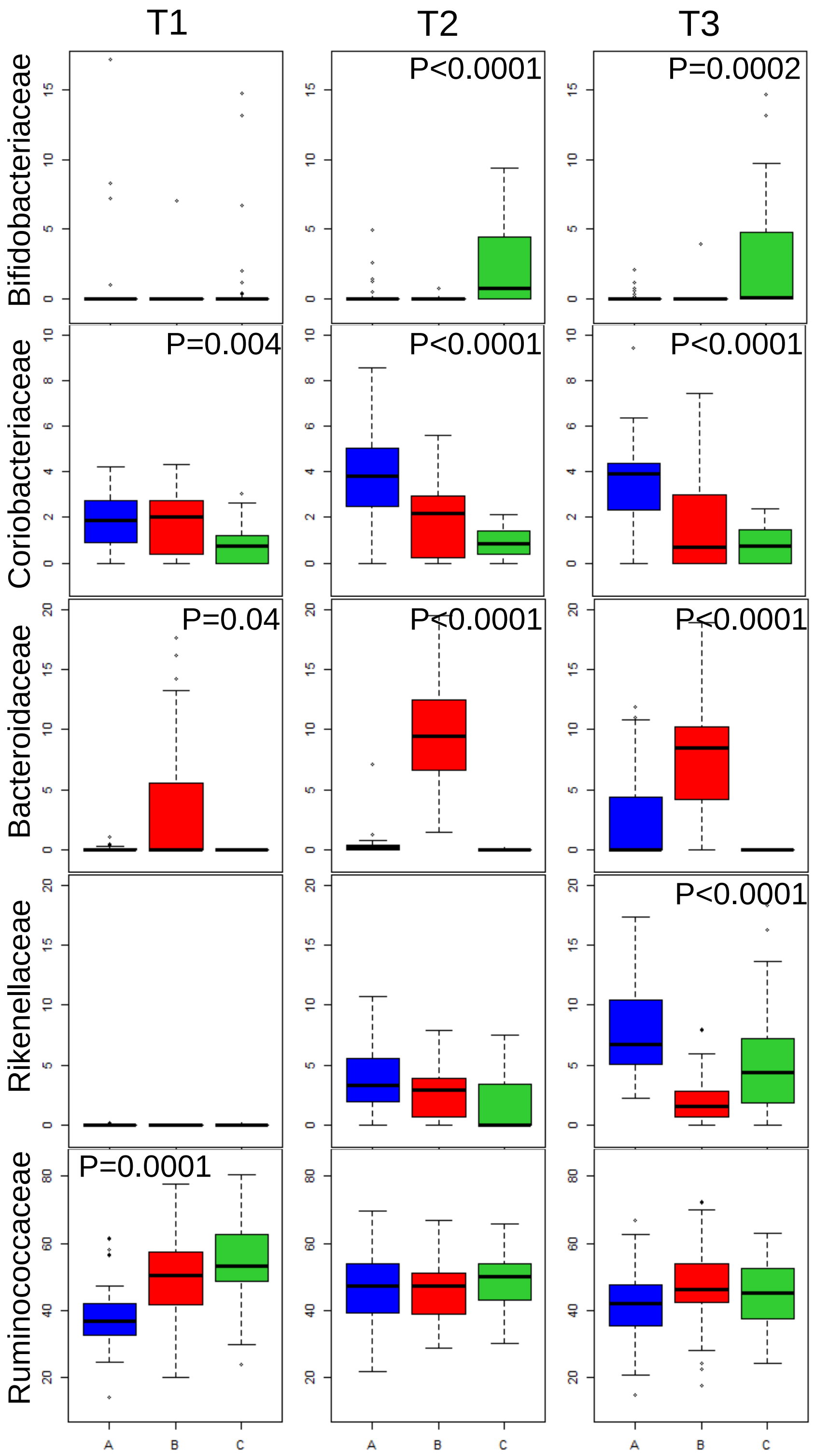

Supplementary Figure S5: Relative abundance distributions of bacterial families in the caecal microbiota of broilers. Box and whiskers distributions of relative abundances (%) in all samples, at the three time points (from left to right) are depicted for those families showing significant differences between the three group (A, blue; B, red; C, green) in at least one timepoint. Bejamini-Hocherg corrected P values obtained from Kruskall-Wallis test are reported when statistical significance was reached (P<0.05).

Supplementary table S1. Feed composition (%) in relation to different feeding phases (starter, from 0 to 15 days; grower, from 16 to 28 days; finisher, from 29 to 42 days).

INGREDIENTS	STARTER	GROWER	FINISHER
Corn	42.20	37.50	37.50
Wheat	10.18	20.38	25.47
Soybean meal	18.56	18.57	10.13
Fullfat soybean	12.22	15.28	17.32
Soybean concentrate high cp	5.09	0.00	0.00
Sunflower	2.04	2.04	3.05
Gluten corn	3.06	0.00	0.00
Vegetable oil	3.18	3.38	4.27
Dicalcium phosphate	1.24	0.88	0.57
Calcium carbonate	0.64	0.50	0.43
Salt	0.26	0.24	0.16
Sodium bicarbonate	0.10	0.10	0.21
Choline cloride	0.10	0.10	0.05
Lisine sulphate	0.54	0.44	0.41
DL methionine	0.39	0.39	0.29
Threonine	0.14	0.14	0.08
Xylanase 0.05%	0.06	0.06	0.06
Total	100.00	100.00	100.00

Supplementary Table S2. Vitamin B2 target and analyzed content in feed for each group, (mg/kg of feed and % vs target), in the different feeding phase diets.

	GROUP A	GROUP B	GROUP C
**TARGET LEVEL**	**5**	**50**	**100**
**ANALYZED**			
STARTER	7.30 (146)	42.2 (84,4)	114 (114)
GROWER	4.66 (93,2)	47.8 (95,6)	112 (112)
FINISHER	5.74 (115)	52.7 (105,4)	102.8 (102,8)
